# Engaging study participants in interpreting results: lessons from the TRIO study in Kenya and South Africa

**DOI:** 10.2147/IJWH.S193905

**Published:** 2019-07-12

**Authors:** Kawango Agot, Alexandra M Minnis, Kgahlisho Manenzhe, Erica N Browne, Khatija Ahmed, Timothy Okello, Ariane van der Straten

**Affiliations:** 1 Impact Research and Development Organization , Kisumu, Kenya; 2 Women’s Global Health Imperative, RTI International, San Francisco, CA, USA; 3 Setshaba Research Centre , Soshanguve, South Africa

**Keywords:** end-user research, MPT products, community-engaged research, biomedical HIV prevention

## Abstract

**Background:** Women account for 56% of new HIV infections in sub-Saharan Africa. Multipurpose Prevention Technologies (MPTs) are promising interventions because they combine HIV prevention with a less stigmatizing indication, such as pregnancy. We conducted a study with three placebo-only MPT products in Kisumu, Kenya and Soshanguve, South Africa, to assess preferences for attributes of tablets, vaginal rings and injectable products for dual prevention of HIV and pregnancy (TRIO Study). Here, we present former TRIO participants’ views on the study results.

**Methods:** After study completion in 2017, we held five dissemination sessions (two in Kisumu and three in Soshanguve) and five one-on-one sharing sessions in Soshanguve. Key results were discussed, with a focus on why some study products were more popular than others, which findings were surprising and why some women changed products over time. A thematic approach was used for analysis.

**Results:** All 277 TRIO participants were telephoned, 168 (60.6%) were reached and 117 (42.2%) attended the dissemination sessions: 71 in Kisumu and 46 in Soshanguve. Participants were engaged and interested in the TRIO findings and willingly shared their perspectives and views candidly. Ease of use, discretion and familiarity were highlighted as drivers of product choice whereas novelty presented a challenge. In explaining the discrepancy between preference ratings and choices, participants cited features such as tablets being easy to explain to a partner or to discontinue. In explaining why 20% of participants switched products after practical experience, issues related to relationships with partners and product attributes perceived as unfavorable were paramount.

**Conclusion:** The dissemination sessions provided an important forum for study participants to interrogate and explain the results to minimize possible misinterpretation. This exercise helped give context to the results, ensured correct lessons were derived from those results and increased credibility of the findings reported by the investigators.

## Introduction

HIV infection in Eastern and Southern Africa accounted for 43% of global HIV incidence in 2016.^1^ In 2015 in sub-Saharan Africa (SSA), women accounted for 56% of the new infections.^2^ Despite the disproportionate burden of HIV among women, prevention options are limited, particularly for adolescent girls and young women.^3^,^4^ Biomedical interventions such as oral pre-exposure prophylaxis (PrEP), vaginal gel and vaginal ring have shown mixed results across SSA. For instance, due to suboptimal adherence, PrEP showed no protective effect among women in clinical trials,^5^,^6^ intermittent dosing of tenofovir vaginal gel also showed no protection,^7^ as did vaginal ring among younger women (<21 years).^8^,^9^ In contrast, where adherence was high, oral PrEP was effective in reducing the risk of acquiring HIV;^10^,^11^ similarly, the vaginal ring was effective in women ages ≥22 who had objective evidence of ring use.^9^,^10^

One way to address this adherence challenge with biomedical HIV prevention interventions is to co-formulate products that combine HIV prevention with something less stigmatizing but in high demand, such as contraceptives or prevention of sexually transmitted infections (STIs).^3^,^12^ Capturing the views of potential end-users early in the product development process is a growing interest among researchers to gain insights that will yield products most likely to have high acceptability and use.^13^ The user-centered approach to product development calls for ensuring that results from studies conducted to inform product design are interrogated by those who participated in the study for correct interpretation. Previously, participants have decried the lack of or inadequate dissemination of study results to those who took part in the studies.^14^,^15^ Often, data are analyzed and interpreted by research teams and disseminated mainly through publications in academic journals, presentations at scientific conferences and press releases to the media^16^,^17^ without soliciting participant input on their interpretation. Such results are open to misinterpretation as the research teams, principal investigators and research leadership who live outside of the researched communities providing product design input may lack contextual knowledge of people living within the community that is key to understanding community feedback, especially for unexpected findings.^15^ Sharing results with study participants and getting their feedback on and reaction to the results therefore improves the likelihood of correct and more nuanced interpretation and application of the findings.^18^

We carried out a study in Kisumu, Kenya, and Soshanguve, South Africa on the acceptability of and preferences among three placebo-only Multipurpose Prevention Technology (MPT) products (monthly vaginal ring, monthly injections and daily tablet) for the future prevention of HIV and pregnancy.^19^–^21^ Here, we present results of discussions held during dissemination sessions convened to present the key results to former study participants, gather their views on the results and help the study team understand and interpret the key results, especially those that were unexpected.

## Methods

The study, known as TRIO (Tablets, Ring, Injections as Options), was conducted in Kisumu, Kenya, by Impact Research and Development Organization, and in Soshanguve, South Africa, by the Setshaba Research Center, in partnership with the RTI International’s Women’s Global Health Imperative, a research program based in San Francisco, California, United States. The study obtained ethics approval from Scientific and Ethics Review Unit of the Kenya Medical Research Institute (Ref #: NON-SCC 474) and from South Africa’s Pharma Ethics (Pty) Ltd (Ref #: 150110905). RTI International’s IRB transferred oversight responsibilities to the Kenyan and South African IRBs through formal authorization agreements. All participants provided written informed consent prior to study enrolment. This study was conducted in accordance with the Declaration of Helsinki. The study was not registered with a clinical trials site as the products were all placebo.

### Highlights of the parent study

Detailed TRIO methods and key findings have been described elsewhere.^19^,^21^ Briefly, the main goal was to find out which of three placebo delivery forms young women would prefer for prevention of both HIV and pregnancy. For each product, and at different stages of the study (eg, prior to trying the products, after trying each for one month and finally after 2 months of use of their chosen product), participants were asked what aspects of the products they liked and disliked, and what could be done to address the dislikes. The study was designed to assess the level of preference for each product in terms of physical attributes, dosage and route of administration.

**Figure 1:**
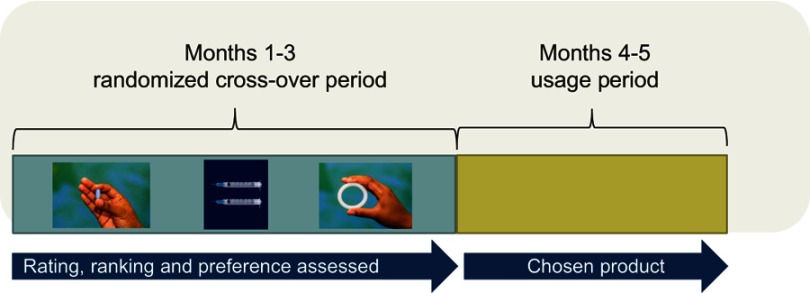
TRIO study design. **Abbreviation:** TRIO, Tablets, Ring, Injections as Options.

Altogether, 277 women ages 18–30 years joined the main clinical study, 137 at the Kisumu site and 140 at the Soshanguve site. At enrollment, participants were first asked for their opinions about each product after viewing photos with no other explanation. They were then shown an educational video demonstrating how each product is used, after which they were again asked for their opinions. In stage 1 of the clinical study, participants were randomized to the order for trying each of the three products.^21^ After using each product for a month, they were given the option to choose a preferred product to use for additional two months (usage period, stage 2), with the option to change to a different product at midpoint (Figure 1). As part of risk reduction counseling participants were also provided with male condoms at each visit.

In the dissemination sessions, we presented key results to participants, with pre-set questions to guide the discussion (Table 1). Participants were taken through a presentation of the key results and then discussed each question.

**Table 1:** Summary of select results and discussion questions

Key study results	Discussion questions after presenting the results
Question topic: How the products were rated before and after watching a short video on each product using a 1–5 point Likert scale (low=1) Result: Mean ring rating before=2.4 and after=2.8, *p*<0.001; injection rating before=3.7 and after=3.8, *p*=0.10; tablet rating before=3.2 and after=3.2, *p*=0.14^20^	Why do you think ratings changed more for the ring after watching the video but not for the injections or the tablets?
Question topic: How participants rated the products after watching the video vs after using each for a month Result: Ring after video=2.8, after use=3.7, *p*<0.001; injection after video=3.8, after use=4.4, *p*<0.001; tablet after video=3.3, after use=3.5, *p*=0.013^20^	Why do you think more people chose the tablets (20.9%) compared to ring (14.9%) though the average rating of the tablet was lower compared to the ring?
Question topic: Which Tablets, Ring, Injections as Options (TRIO) product was most preferred (ranking exercise) Result: When condom was added as an option, 10–20% of participants preferred condom to the 3 products.^21^	Why did some women in both countries prefer condoms more than the 3 products?
Question topic: Which participants switched their products at midpoint in stage 2 and what were their reasons for switching Result: 50 participants (20%) switched, and of those, the following switches were made:21 (i) Of those who first chose the ring (n=37) Ring to injection: n=7, 19% Ring to tablet: n=5, 14% (ii) Of those who first chose tablets (n=52) Tablet to injection: n=9, 17% Tablet to ring: n=2, 4% (iii) Of those who first chose injections (n=160) Injection to ring: n=10, 6% Injection to tablet: n=17, 11%	(i) In stage 1, women tried each product for a month so why do you think they switched after choosing the product to use in stage 2? (ii) Was one month long enough to try each product and form a full opinion of it?
Question topic: What was the participant reaction to the products women chose at stage 2 Result: Product choices at stage 2: Injection 64%, ring 15% and tablets 21%.	What did you find surprising and what came out as you expected?

At both sites, all participants were called using the telephone contacts given when they enrolled in the study. The Kisumu site held two large group sessions (comprised of 33 and 38 participants) while the Soshanguve site held three group sessions that drew 41 participants and one-on-one sessions with five participants who were unable to join the group sessions. These Soshanguve one-on-one sessions were held in a similar manner to the group sessions to ensure the dissemination methods across sites did not vary considerably. All dissemination sessions were facilitated by the study coordinator for each site. At the Kisumu site, the sessions took place at an off-site hotel and participants contributed to the discussion in a language of their choice (English, Kiswahili, Dholuo or a mixture of the three languages); however, because the site did not seek ethics approval to audio-record the session proceedings, the notes were taken in English by three staff and expanded immediately after the sessions. In order to accurately record contributions, participants were asked to repeat the responses that were deemed suitable for direct quotation so they could be captured verbatim. At the Soshanguve site, the sessions were held within the study facility, notes were taken by the facilitator, in addition to audio-recording of the sessions. These were transcribed and translated from seTswana into English.

### Data analysis

The transcripts from South Africa and expanded notes from Kenya were typed and manual content analysis performed. The lead author (KA) identified key themes from the notes using the questions (Table 1). The themes were reviewed by KM and the principal investigator (AVS) and modifications suggested were discussed and final themes agreed on by the three of the authors (Box 1). KA used these themes to manually analyze the contents of the transcripts and notes and identified representative quotes where relevant.

**Box 1:** Key qualitative themes

Reasons ratings changed most for the ring after watching the video compared to injection or tablets.
Reasons for rating tablets lower than ring yet preferring it when given a choice to select between the two.
Reasons some women preferred condoms to the 3 products.
Reasons some women switched products after choosing the product to use in stage 2. Adequacy of duration of trying products.
Study results that were surprising and those that were expected.

## Results

Of 277 former TRIO participants contacted to participate in a dissemination session, 117 (42%) attended one: 71 of 137 participants (52%) in Kisumu and 46 of 140 participants (33%) in Soshanguve. Table 2 shows key demographic characteristics between those who attended the sessions and those who did not. Besides differences in casual sexual partner and ever exchanged sex (Kisumu) and completing secondary school and completing the study (Soshanguve), other characteristics examined, including choice of product, did not differ by site or attendance to the session.

**Table 2:** Comparison of participant characteristics between those who did and did not attend a TRIO study dissemination session, by country

	Attended Dissemination Session							
	Kenya N=137				South Africa N=140			
Yes		No		Yes		No	
	N	(%)	N	(%)	N	(%)	N	(%)
Total	71	(100)	66	(100)	46	(100)	94	(100)
Median age years, (IQR)	23	(21–26)	22	(20–26)	23	20–26	23	21–26
18–24	45	(63)	46	(70)	33	(72)	59	(63)
25–30	26	(37)	20	(30)	13	(28)	35	(37)
Currently have a primary partner	67	(94)	59	(89)	45	(98)	90	(96)
Married or cohabiting	38	(54)	28	(42)	5	(11)	8	(9)
Currently have a casual sex partner^1^	9	(13)	22	(33)	5	(11)	14	(15)
Exchange sex ever^1^	7	(10)	18	(27)	2	(4)	6	(6)
Parity >0	58	(82)	49	(74)	36	(78)	73	(78)
Completed secondary school^2^	28	(39)	29	(44)	34	(74)	52	(55)
Earns an income	34	(48)	34	(52)	5	(11)	13	(14)
Completed study^2^	67	(88)	55	(83)	45	(98)	79	(84)
Choice product								
Tablets	20	(28)	13	(20)	8	(17)	11	(12)
Ring	11	(16)	9	(14)	3	(7)	14	(15)
Injections	36	(51)	34	(52)	34	(74)	56	(60)
NA	4	(6)	10	(15)	1	(2)	13	(14)
NA = Not applicable, did not complete month-3 visit								
1 Kenya: *p*<0.05								
2 South Africa: *p*<0.05								

By way of introduction, participants in both sites were asked what they remembered about the study. They demonstrated clear understanding of the three products, including that these were placebos. At the Kisumu site, participants explained that: “Two injections were given that last for one month, to prevent HIV infection and pregnancy [assuming active products];” another said: “One tablet was taken once a day;” and one added: “A ring was inserted in the vagina which lasted 30 days.” When asked about what the products comprised of, a participant from Kisumu responded: “None of the products had any drugs, they were placebos used to understand which products the women would like to use,” and one from Soshanguve gave a similar explanation: “It is like the placebo, it was not the real one at that time.”

We analyzed and present five key topics pertaining to TRIO study results on which we asked participants to give their views, suggestions and reactions (see Table 1):

## Why ratings changed most for the ring after watching the video at enrollment visit

One interesting finding was that after watching the educational video, participants’ reported interest in the ring increased while their rating of the tablets and injections remained largely the same. Interpretations that were common in all sessions at both sites included participants being familiar with injections and tablets; hence, they did not learn anything new about them from the video. Specifically, they reported that the educational video session taught them “about the new product and allayed their initial fears” thus giving them a more positive attitude towards the ring. One participant from Kisumu said: “We were comfortable with the injection and tablets but the ring was something new. We feared the size and putting it in, but after watching video we became positive about the ring”. For others, learning from the video that “the ring is put in there once and for all” was the reason the rating improved. Participants further explained that fears such as ring disappearing in their bodies, getting expelled out of their vagina or getting felt by the partners during sex were addressed in the video thus improving the rating.

## Why more participants chose the tablets compared to ring at entry into stage 2

Another unexpected result was that more participants chose tablets than ring for stage 2 even though the average rating for the tablet during stage 1 was significantly lower than the ring on a 5-point Likert scale (1=low; see Table 1). Kisumu participants believed that more people would have taken the tablets home with no intention of using them unlike the injection which was provider-administered, or the ring which was inserted at the clinic and examined visually after a month to evaluate whether it had been used (a study procedure about which participants were informed): “They picked the tablets but they did not swallow because there’s no evidence unlike the injection they were getting at the clinic or ring that we were told there was evidence of use”.

Other participants gave various explanations, including: tablet was perceived to be safe because one could stop taking them any time whereas women still had lingering concerns about the safety of the ring “while in there for a month” or injections “which you cannot remove [reverse]”. One participant said that the process of ring insertion at the clinic – which was a study procedure requirement – was embarrassing, making some participants opt for tablets: “The tablet scored because one tablet is taken once a day and it’s easy to swallow while the discomfort of ring insertion is not welcome. The insertion process of opening your legs to a stranger to check your private part was embarrassing” (Soshanguve site).

Some participants linked product choice to relationship considerations and disclosure: “For those participants who had not revealed their participation to their partners, the tablet was more preferred compared to the ring because it was easy to explain [away] as ‘just another tablet from the clinic’ but it would be more difficult to explain [away] the ring if the partner found out.” (Kisumu site) Another participant agreed, adding: “With the tablet you can say that you are on medication for a cough or something while with the ring you lack words to explain when you are ‘caught’ because your spouse is not aware of the function.”

## Why some women preferred condoms more than the three products

At the end of stage 1 in the clinical study, 10–20% of participants reported preferring condoms compared to the TRIO products. Commenting on this finding, several participants stated that condoms are advantageous because they also prevent STIs unlike the TRIO products that would prevent HIV and pregnancy only. Another attribute deemed attractive about condoms was the convenience of needing to use only during sex: “Condom is only used when required but medication you have to use every day like injection and tablet, ring is there every day even when you do not need it” (Kisumu site). Further, others observed that condoms have few or no side effects unlike products tested in TRIO that could have side effects once active drugs are added. Similar to sentiments expressed for preference for tablets relative to ring, some participants argued that it is easier to talk to a sexual partner about condoms than the less familiar TRIO products.

## Why one-fifth of the women switched products mid-point in stage 2

Participants had a “try out” month to use each product before being given an opportunity to choose a preferred product at entry into stage 2 for two months of usage period (Figure 1). They had the option to change to a different product at midpoint into the usage period if they wished, and 20% switched. The participants offered multiple explanations for switching products. For some, those who did not disclose product use to their partners may have felt that using it for 2 months was too long without the partner realizing, so opted to change: “There are those who at the beginning the partners did not know they were in the study so using the tablet long term (2 months) was not easy, therefore they changed to injection which was more private to use” (Kisumu site). Other participants argued that changes in relationship status could have influenced the switch of products; for instance, a participant who had opted for a method such as a vaginal ring could have switched to a more discreet product such as injection after getting married or entering into a new or stable relationship. The switch was also ascribed to product attributes perceived as unfavorable. One woman who had switched to injection said: “I changed from tablet to injection due to privacy and not wanting my partner to realize I am on a product” (Kisumu site). For others, a change from tablet to injection was precipitated by challenges with adherence to daily ingestion. For the ring, one mentioned that the discomfort with insertion prompted the switch: “I changed from ring to injection because the ring I did not know how to properly put it in so it hurt and I got stomach cramps” (Kisumu site).

Given that one-fifth of the participants switched products, we also inquired whether they felt that the one month they were given to use each product during stage 1 was adequate to form an opinion on which product they preferred and would use for stage 2 (the usage period) (Figure 1). Most participants in both sites felt that one month was sufficient to determine a product of choice although a few suggested that 2, 3 or even 6 months per product would have been valuable to afford opportunity to better understand the products and form an opinion. One woman from Kisumu pointed out: “I need the first month to see if there are side effects and the second month to make a decision.” Some South African participants were specific that being new, the ring required tryout for a longer time to form a considered opinion: “I think 2 months is going to be better because…I was going to get used to it and even forget that I have inserted it.”

A comment expressed multiple times in Soshanguve but not in Kisumu was that the products should have had the medication so that they can test the active pharmaceutical ingredient and know the side effects real products will have: “TRIO products should not have been placebos because participants would have loved to feel the real side effects and make a decision of which product they liked or disliked considering the product’s side effects too.”

## Reactions to the results – what was expected and what was surprising

Reactions to the results varied between the two sites. There was overwhelming agreement by participants in Kisumu that the findings came out as expected, specifically that the injection was the most popular product followed by tablets and that the ring was least popular. There was a near-consensus view that the results were a true representation of women in the various communities where they came from. Participants from Soshanguve, however, had mixed reactions: while among those who came to individual dissemination meetings agreed that participants preferred injection because it is easy to use and is only taken once a month, those who attended the sessions in a group were rather disappointed that the injection was favored over the ring, which they expected to “win”.

A minority of participants in Kisumu were also surprised that the ring was the least chosen, given that many participants liked the ring on initial use (reflected in the higher rating for the ring): “I had expected the tablets to be the least preferred as they are big in size, look like ARVs and are cumbersome to carry around when travelling while the ring is painless to use and if placed correctly cannot be felt by a sexual partner.” Another Kisumu participant said: “I thought the ring would win because the tablets cause stigma and the injection is painful but the ring is comfortable.” Several participants from Kisumu wondered why Kenyans prefer the tablets but are poor at swallowing them, asking “what if they were not placebo?” meaning if in TRIO they failed to adhere to placebo that has no side effects, chances are high that their adherence would even be lower if the pills had active drugs.

## Discussion

We held TRIO study dissemination sessions in Kisumu and Soshanguve to share results with former participants, obtain their views on the key findings and engage them throughout the full research study lifecycle. Participants were interested in the findings and willingly shared their perspectives and views. Lack of familiarity with a “new” product like the ring, coupled with difficulty associated with the mode of application, were found to be more challenging to overcome and will require extra education and possibly practice, with ongoing lingering concerns that may undermine acceptability of new dosage forms. Similarly, discreet and infrequently administered products like injection were more attractive to end-users. Switching between products during stage 2 may reflect “consumer behavior” resulting from having options and exercising choice, and not necessarily because of disliking a particular product. Therefore, ease of use, discretion and familiarity, but not novelty, may be a driver of choice.

### Education and experience are important for acceptability of novel products

After watching an educational video on the three products, the ratings improved for the ring but not for injection or tablets, which was attributed to the fact that the ring was novel. This attests to the fact that early education on new products is critical to allay fears and improve acceptability. Additionally, the rating improved further after using each product for a month.^20^ Such incremental improvement in acceptability and uptake of innovations have been demonstrated in other studies. McLellan-Lemal and colleagues^12^ argue that when acceptability is based only on information about and examination of a product, it is less reliable than when it is based on actual experience with a new product. Similarly, a study on acceptability of vaginal ring for HIV prevention observed improved adherence with practice and experience.^22^

### Discrepancy between rating and choice

In the study, we observed a significant gap between choice, stated adherence and evidence of adherence derived from the Wisepill electronic dose monitoring device, which records a date and time stamp each time it is opened.^21^ Participants in the dissemination sessions provided insight that shaped our interpretations and can inform the design of future studies with placebo products. For our purposes, having exposure to the product was more critical that demonstrating adherence. These participant comments point to how stated preference may be more reliable than actual choice if there is no direct benefit to using the product, as in placebo studies. In the future, if monitoring the actual use of a product is an important outcome of the study, there must be an objective measure of use, as any self-report is likely to be inflated. Further, the ring was shunned due to embarrassment associated with insertion at the clinic and fear of partners accidentally feeling it during sex. It is apparent that some of the excitement over a new product and delivery system (the ring) waned over time and users reverted to choosing what was more familiar to them – injection and tablets. Multiple studies in South Africa report similar findings: that participants’ initial enthusiasm with new prevention technologies dissipates when they fully conceptualize what it means to use a product in their daily lives after weighing multiple product attributes in combination.^23^,^24^

### Familiarity, ease of use and discretion

After trying each product for one month, then given an opportunity to select a preferred product and use for 2 months (with an option to change after one month), one-fifth of the participants switched to a different product. One explanation could be the very reason why various biomedical studies are conducted – to provide users with options. We argue that providing many options could, in certain circumstances such as when there is no adverse health effect, make users switch products at will. A Kenyan study exploring acceptability of contraceptive vaginal ring reported that women admitted to switching from one method to another because of availability of a wide range of modern family planning approaches.^12^ While there was no overall effect of product choice on switching at stage 2,^21^ compared to participants who chose injection those who chose the ring had a higher odds of switching, suggesting that participants were attracted to or were looking for a product that combines what is familiar with what is less burdensome. The desire by users to go for familiarity, discretion and ease of use is an indication to product developers on what to consider if they expect higher acceptability and persistence for a given delivery form by end-users.

### Strengths and limitations

One of the strengths of this study is that we obtained candid views of participants after completing the TRIO study, in more informal settings, which likely encouraged greater honesty and spontaneous responses than during a study visit where greater social desirability may be at play. Using this approach to minimize social desirability bias was reported in follow-up studies to FEM-PrEP and VOICE-D conducted to explore the reasons for the low adherence during the trial.^25^,^26^ In addition, engaging participants to help in providing the context of the study results before publications improves their credibility.15 Despite these strengths, the study had a major limitation. While we reached out to all participants, only 42% attended the dissemination sessions, with variation by site. This may reflect site differences in reimbursement offered to participants (transportation costs and time compensation provided separately in Kisumu whereas transportation costs only in Soshanguve) or other logistical factors pertaining to when the workshops took place (e.g., weekends vs weekdays). It is therefore possible that these participants had different opinions from the majority. However, those who attended the sessions and those who did not were comparable in most characteristics, most importantly in product choice. In addition, familiarity with the study coordinators at each site could have introduced social desirability bias in responses, although the apparent candidness during the discussions suggests that participants felt comfortable sharing product dislikes and challenges encountered and regarded their role as advisors to the study team rather than feeling expected to deliver a message that would be regarded as favorable.

## Conclusion

The dissemination sessions provided an opportunity to present the results to former TRIO study participants and hear their views on the key findings. That over 40% of former participants attended the sessions six months after exiting the study highlighted their keen interest in learning of study results and having an opportunity to discuss them with the study team. Engaging participants in interpreting results of studies they participate in therefore helps in giving context to the results and ensuring correct lessons are derived from those results.
